# Calbindin D28k-Immunoreactivity in Human Enteric Neurons

**DOI:** 10.3390/ijms19010194

**Published:** 2018-01-08

**Authors:** Katharina Zetzmann, Johanna Strehl, Carol Geppert, Stefanie Kuerten, Samir Jabari, Axel Brehmer

**Affiliations:** 1 Institute of Anatomy and Cell Biology, University of Erlangen-Nuremberg, Krankenhausstraße 9, D-91054 Erlangen, Germany; katharina.zetzmann@gmx.de (K.Z.); stefanie.kuerten@fau.de (S.K.); samir.jabari@fau.de (S.J.); 2 Institute of Pathology, University of Erlangen-Nuremberg, Krankenhausstraße 8-10, D-91054 Erlangen, Germany; johanna.strehl@uk-erlangen.de (J.S.); carol.geppert@uk-erlangen.de (C.G.)

**Keywords:** calcium binding protein, calretinin, enteric nervous system, morphology, myenteric plexus, submucosal plexus

## Abstract

Calbindin (CALB) is well established as immunohistochemical marker for intrinsic primary afferent neurons in the guinea pig gut. Its expression by numerous human enteric neurons has been demonstrated but little is known about particular types of neurons immunoreactive for CALB. Here we investigated small and large intestinal wholemount sets of 26 tumor patients in order to evaluate (1) the proportion of CALB^+^ neurons in the total neuron population, (2) the colocalization of CALB with calretinin (CALR), somatostatin (SOM) and vasoactive intestinal peptide (VIP) and (3) the morphology of CALB^+^ neurons. CALB^+^ neurons represented a minority of myenteric neurons (small intestine: 31%; large intestine: 25%) and the majority of submucosal neurons (between 72 and 95%). In the submucosa, most CALB^+^ neurons co-stained for CALR and VIP (between 69 and 80%) or for SOM (between 20 and 3%). In the myenteric plexus, 85% of CALB^+^ neurons did not co-stain with the other markers investigated. An unequivocal correlation between CALB reactivity and neuronal morphology was found for myenteric type III neurons in the small intestine: uniaxonal neurons with long, slender and branched dendrites were generally positive for CALB. Since also other neurons displayed occasional CALB reactivity, this protein is not suited as an exclusive marker for type III neurons.

## 1. Introduction

The combined application of various neuroscientific methods has enabled the identification and characterization of different types of enteric neurons primarily in the guinea pig [1,2]. One of these methods was the immunohistochemical distinction between several enteric neuron types by different markers that deciphered their chemical codes. The value of these chemical codes consists of the relatively simple possibility of representing neuron types in gut tissue samples, e.g., under experimental or pathological conditions. This allows us to draw conclusions about possible selective changes in neuron populations, i.e., a neurohistopathological diagnosis, by discriminating between enteric neuron types [3].

In enteric neuroscience, calbindin (CALB) is a “famous” immunomarker since antibodies against this calcium binding protein selectively label most morphological type II neurons, the intrinsic primary afferent neurons in the guinea-pig small intestine (IPANs) [4,5,6,7].

Although CALB is also expressed by a substantial number of human enteric neurons (counted in the duodenum [8]), immunohistochemical staining of CALB succeeded only in a few myenteric type II neurons [9]. We have shown that human myenteric type II neurons, the putative myenteric IPANs, are immunohistochemically characterized by the colocalization of calretinin (CALR) with somatostatin (SOM) [10,11].

The human enteric nervous system consists of three ganglionated (see below) and several non-ganglionated plexus (e.g., in the muscle layers, the mucosa, etc. [12]). The ganglionated myenteric plexus lies between the circular and longitudinal muscle layers. Human submucosal neurons are located within two ganglionated subplexus [12]. The external (or outer) submucosal plexus (ESP) is located under the inner border of the circular muscle layer and is mostly monolayered. The internal (or inner) submucosal plexus (ISP) occupies the inner half of the submucosa and is frequently two- or even three-layered [3,12]. As to the distribution patterns of neuron types within both plexus there are, as far as we know, only quantitative differences between them. Two larger submucosal neuronal populations are known to date [13]. One is non-dendritic, (pseudo-) uniaxonal and immunoreactive for SOM and, partly, substance P [14]. The other one displays a multidendritic appearance and is immunoreactive for vasoactive intestinal peptide (VIP) and, partly, CALR [15]. Both submucosal neuron types are cholinergic and project into the mucosa. Additionally, there are some submucosal nitrergic neurons, which are, however, occasionally absent [15]. 

Thus, immunohistochemistry for the calcium binding protein CALR has been proven to be useful for the identification of particular human enteric neuron types: in the myenteric plexus (MP), in colocalization with SOM, it labels morphological type II neurons, the putative IPANs; in the submucosal plexus it is frequently colocalized with VIP and a marker for multidendritic neurons, which are putative mucosal effector neurons (see above). Colocalization of CALR and VIP in these neurons was almost complete in the colon but not in the small intestine [15].

The aim of this study was to address the question whether another calcium binding protein, namely CALB, may also be a useful immunohistochemical marker for identifying a particular human enteric neuron type. To this end, we quantified the proportion of CALB^+^ neurons in relation to the putative total enteric neuronal population throughout all small intestinal and colonic subregions, which was stained by the pan-neuronal marker HU C/D (HU) [16]. Furthermore, we estimated the colocalization rates of CALB^+^ neurons with CALR, VIP and SOM: in the human MP colocalization of CALR and SOM label the putative IPANs [10,11]; in the human submucosal plexus VIP and SOM label the two larger different neuron populations known so far [14,15]. Finally, we analyzed the morphology of CALB^+^ neurons by co-staining with neurofilament (NF) in the myenteric plexus [7,17] and by peripherin (PERI) in the two submucosal plexus [12,13].

## 2. Results

### 2.1. Wholemounts Stained for HU C/D (HU) and Calbindin (CALB)

**Figure 1:**
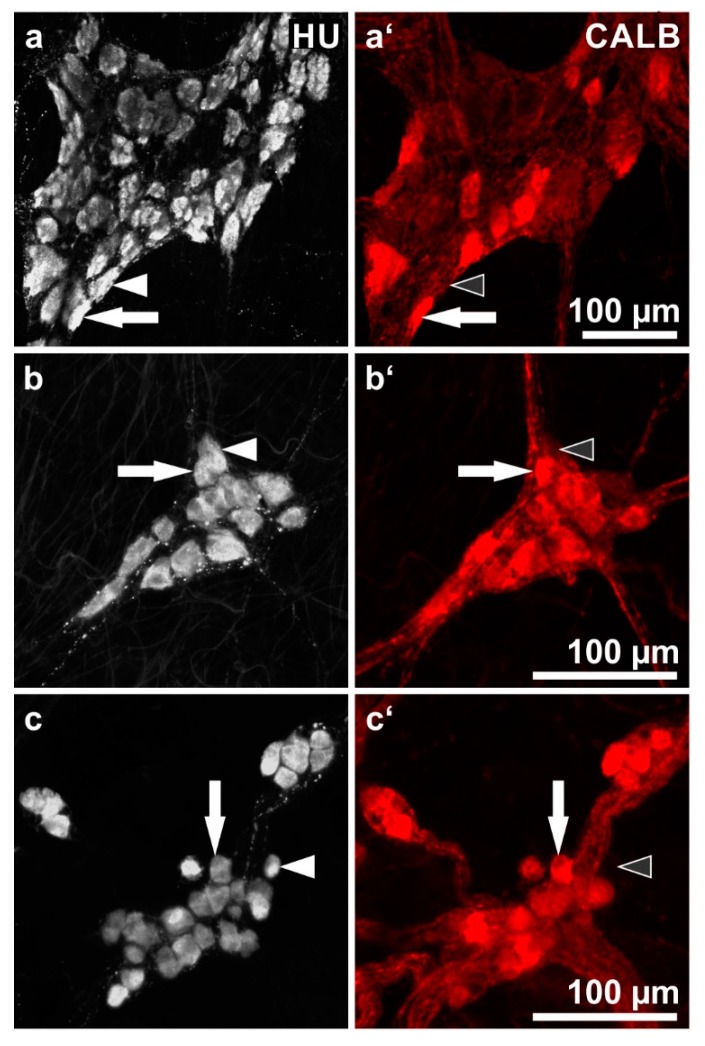
Human enteric ganglia double immunostaining for HU C/D (HU; grey) and calbindin (CALB; red): (**a**,**a’**) myenteric ganglion, (**b**,**b’**) external submucosal ganglion, (**c**,**c’**) internal submucosal ganglia. Pairs of **filled arrows** point at representative neurons positive for both HU and CALB; pairs of **arrowheads** (filled or empty, respectively) point at HU^+^ but CALB^−^ neurons. (Patients data: (**a**) 70 years, ileum, male; (**b**,**c**) 70 years, sigmoid colon, female).

This staining combination allows for an estimation of the proportion of CALB^+^ neurons in relation to the whole enteric neuron population (Figure 1, Table 1).

Overall, in both the small intestine and the colon, CALB^+^ neurons represented a minority in the MP and the majority in the two submucosal plexus. The intensity of fluorescence labeling varied considerably, some neuronal cell bodies were intensely fluorescent but others displayed weak labeling.

In the MP of the small intestine, the proportions varied between 25.1 and 36.7% (total 31.1%). In the colon, the values ranged between 21.1 and 30.5% (total 25.2%).

In the ESP and ISP of the small intestine, the proportions ranged between 56.8 and 86.3% (total ESP: 81%; ISP: 72.3%). In the colon, all values were higher than 90%, 93.1% in the ESP and 95% in the ISP.

### 2.2. Wholemount Quadruple Staining for Calbindin (CALB), Calretinin (CALR), Somatostatin (SOM) and Vasoactive Intestinal Peptide (VIP)

**Figure 2:**
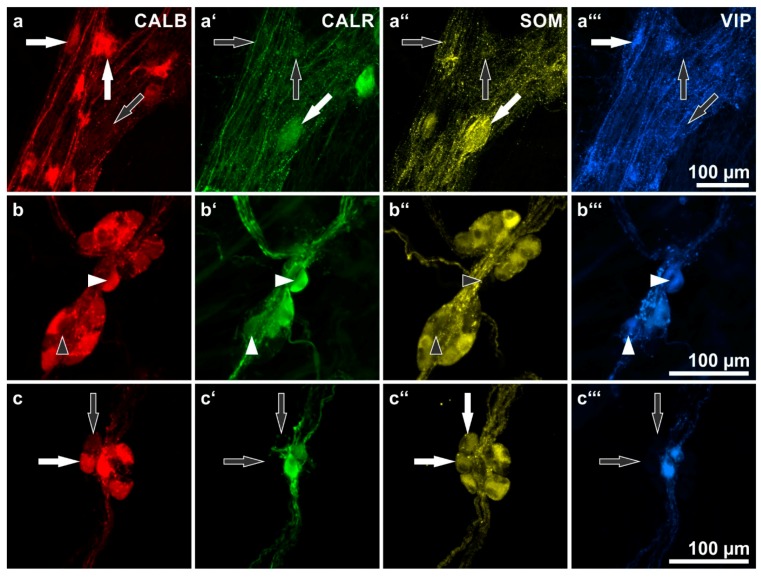
Human enteric ganglia quadruple immunostaining for calbindin (CALB: **a**,**b**,**c**), calretinin (CALR: **a’**,**b’**,**c’**), somatostatin (SOM: **a’’**,**b’**’,**c’’**) and vasoactive intestinal peptide (VIP: **a’’’**,**b’’’**,**c’’’**). (**a**) Myenteric ganglion. **Arrows** point at three representative neurons displaying different combinations of immunolabeling. **Horizontal arrows** indicate a neuron positive for CALB and VIP but negative for CALR and SOM; **vertical arrows** show a neuron positive for CALB only; **oblique arrows** point at a neuron positive for CALR and SOM but negative for CALB and VIP. (**b**) External submucosal ganglion. **Arrowheads** point at two neurons positive for both VIP and CALR but negative for SOM, one neuron is positive for CALB (**horizontal**, **filled arrowhead** in (**b**), the other negative (**vertical**, **empty arrowhead** in (**b**). (**c**) Internal submucosal ganglion. Arrows point at two neurons positive for SOM but negative for VIP and CALR, one neuron is positive for CALB (**horizontal**, **filled arrow** in (**c**), the other one negative (**vertical**, **empty arrow** in (**c**). Patients data: (**a**) 69 years, ascending colon, female; (**b**,**c**) 76 years, duodenum, male.

Here, colocalizations of CALB with CALR, VIP, and/or SOM immunoreactivities in both myenteric and submucosal neurons were studied (Figure 2, Table 2).

Most myenteric CALB-positive neurons did not co-stain for the other markers that were investigated. In the small intestine (total 86%), the proportions of these neurons varied between 96.9% (duodenum) and 79.9% (ileum). In the large intestine (total 71.8%), we found proportions between 61% (descending colon) and 84.3% (sigmoid colon). Of the remaining minority of CALB^+^ neurons, most were co-reactive for VIP or CALR or both (total: 10.8% in the small intestine; 27.5% in the colon). Others co-stained for CALR and/or SOM (total: 2.6% in the small intestine; 0.5% in the colon).

Myenteric neurons co-reactive for all four markers were only found exceptionally (altogether seven neurons: five in the small intestine; two in the ascending colon). Furthermore, only one myenteric neuron co-reactive for CALB, SOM and VIP was observed throughout all specimens namely, in a jejunal wholemount. These rare exceptions were not listed in Table 2.

In contrast to the MP, only a minority of submucosal CALB^+^ neurons did not co-stain for other markers (total of ESP and ISP: 3.6% in the small intestine; 5% in the colon). The overwhelming majority of submucosal CALB^+^ neurons co-stained for VIP and/or CALR (total: ESP 76% and ISP 77.5% in the small intestine; ESP 94.7% and ISP 90.4% in the colon). A further, substantial population of CALB^+^ neurons co-stained for SOM (18.1% and 19.7% in the small intestine; 2.7% and 6% in the colon). As to CALB^+^ neurons co-reactive for both SOM and CALR, only in the jejunum (ESP 2.9%; ISP: 9.5%; these mainly occurred in a single submucosal wholemount) and in the ISP of the transverse colon (2.4%) a noteworthy proportion was found.

Altogether 14 submucosal neurons co-reactive for all four markers were detected (four in jejunal specimens; ten in colonic specimens) and one neuron (jejunum) was co-reactive for CALB, SOM and VIP. These rare exceptions were not listed in Table 2.

### 2.3. Wholemount Quadruple Staining for Morphological Analysis

**Figure 3:**
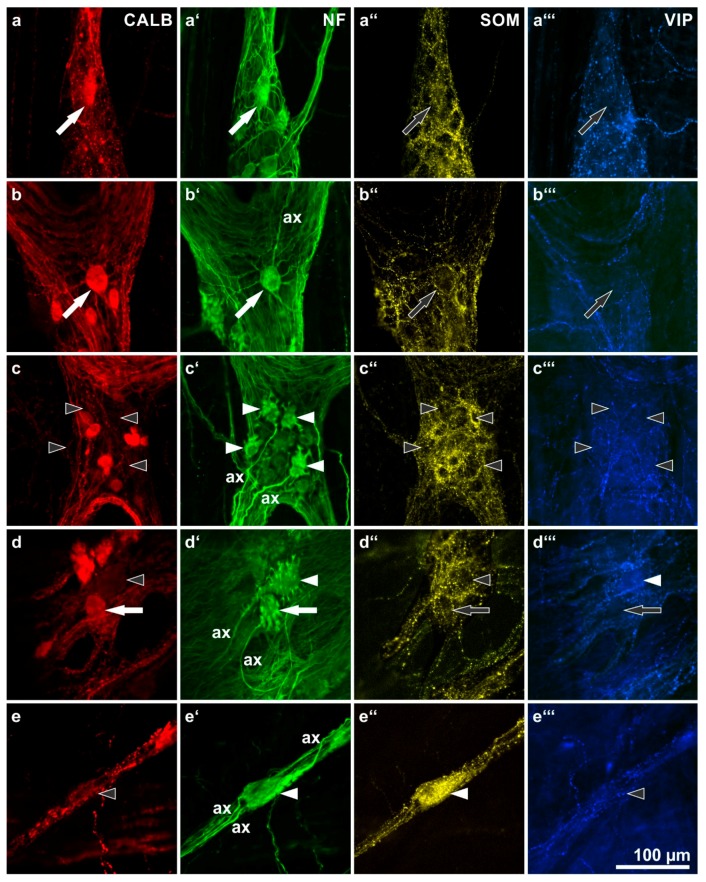
Calbindin (CALB: **a**–**e**) immunoreactivities of morphologically defined, neurofilament (NF: **a’**–**e’**)-labeled human myenteric neuron types and their co-reactivities for somatostatin (SOM: **a’’**–**e’’**) and vasoactive intestinal peptide (VIP: **a’’’**–**e’’’**). (ax = axons of marked neurons). (**a**,**b**) Two type III neurons with long, slender, branched dendrites (**a’**,**b’**: **filled arrows**) positive for CALB (a,b: **filled arrows**) but negative for SOM and VIP (**a’’**,**b’’**: **empty arrows**). (**c**) Four stubby type I neurons (**c’**: **filled arrowheads**) negative for all three other markers (**c**,**c’’**,**c’’’**: **empty arrowheads**). (**d**) A spiny (**d’**: **filled arrowhead**) and a stubby (**d’**: **filled arrow**) type I neuron. The spiny one is negative for both CALB and SOM (**d,d’’**: **empty arrowheads**) but positive for VIP (**d’’’**: **filled arrowhead**), the stubby one is positive for CALB (d: **filled arrow**) but negative for SOM and VIP (**d’’**,**d’’’**: **empty arrows**). (**e**) A non-dendritic type II neuron displaying three axons (**e’**: **filled arrowhead**). It is co-reactive for SOM (**e’’**: **filled arrowhead**) but negative for both CALB and VIP (**e**,**e’’’**: **empty arrowheads**). (Patients data: (**a**–**c**) 59 years, ileum, male; (**d**) 57 years, ascending colon, male; (**e**) 42 years, sigmoid colon, female)

**Figure 4:**
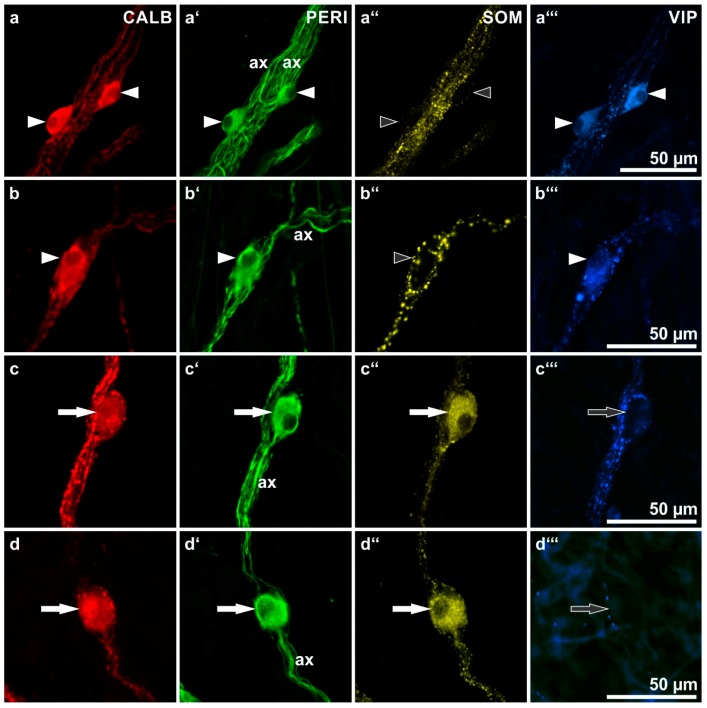
Calbindin (CALB)-immunoreactive human submucosal neurons: shapes as revealed by their peripherin (PERI)-immunoreactivities as well as co-immunolabeling for somatostatin (SOM) and vasoactive intestinal peptide (VIP). (ax = axons of marked neurons). (**a**,**b**) Three neurons (**arrowheads**) displaying a multidendritic/uniaxonal morphology (**a’**,**b’**: **filled arrowheads**) and co-reactivities for VIP and CALB (**a**,**a’’’**,**b**,**b’’’**: **filled arrowheads**) but not for SOM (**a’’**,**b’’**: **empty arrowheads**). (**c**,**d**) Two neurons (**arrows**) displaying a non-dendritic/uniaxonal morphology (**c’**,**d’**: **filled arrows**) and co-reactivities for SOM and CALB (**c**,**c’’**,**d**,**d’’**: **filled arrows**) but not for VIP (**c’’’**,**d’’’**: **empty arrows**). (Patients data: (**a**–**c**) 57 years, ascending colon, male; (**d**) 70 years, duodenum, female).

Next, we focused on a morphological analysis of myenteric neurons co-reactive for both CALB and NF (Figure 3) and of submucosal neurons co-reactive for both CALB and PERI (Figure 4). Mainly smaller myenteric CALB neurons displayed only weak or no reactivity for NF and could not be evaluated morphologically.

In the MP, a distinct correlation between CALB reactivity and morphological features of NF-stained neurons could exclusively be detected for type III-neurons. These had one axon and long, branched, tapering dendrites arranged circumferentially around their soma (Figure 3a,b). They were found only in the small intestinal MP and were generally positive for CALB. Other morphologically defined neuron types were mostly negative for CALB, with a few exceptions each. Stubby type I-neurons (Figure 3c), spiny type I neurons (Figure 3d) or type II neurons (Figure 3e) were frequently negative for CALB. However, in rare cases, neurons of these types were found to be positive for CALB (e.g., a stubby type I neuron in Figure 3d).

In the ESP and ISP no distinct correlation between CALB reactivity and a particular submucosal neuron type could be found. Both multidendritic/VIP-reactive as well as nondendritic/uniaxonal/SOM-reactive neurons frequently displayed coreactivity for CALB (Figure 4) but CALB^−^ neurons of both types were also commonly observed (Figure 2b,c).

### 2.4. Sections Stained for Calbindin (CALB) and Peripherin (PERI)

**Figure 5:**
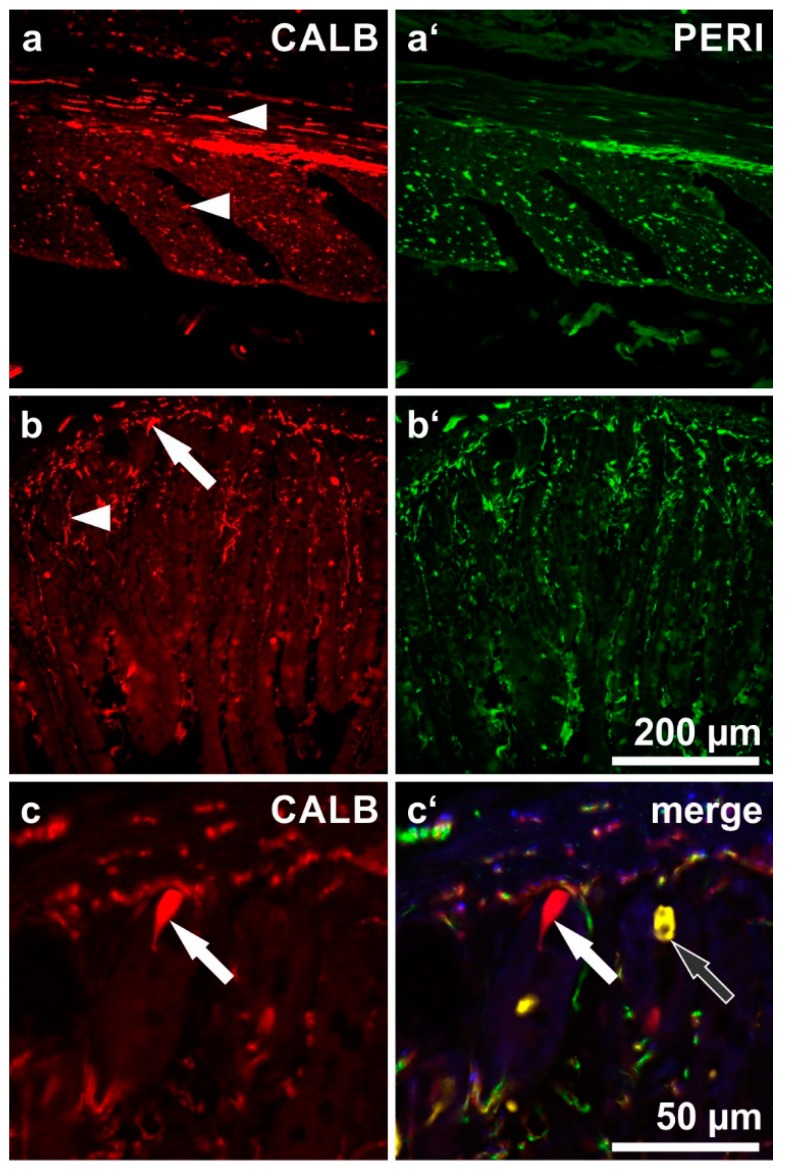
Section through the small intestinal wall, immunostained for calbindin (**a**,**b**,**c**: CALB; red), peripherin (**a’**,**b’**: PERI; green) and somatostatin (yellow;the latter is only depicted in (**c’**). (**a**) CALB-immunoreactive nerve fibers in the longitudinal (**upper arrowhead**) and circular muscle layer (**lower arrowhead**). (**b**) CALB-reactive mucosal nerve fibers (**arrowhead**). The arrow points at an enteroendocrine cell lying in the base of a mucosal crypt and being positive for CALB. (**c**) The same enteroendocrine cell enlarged (**filled arrow**), not far from another endocrine cell reactive for somatostatin (**empty arrow**).

In these specimens we demonstrated the distribution pattern of CALB^+^ nerve fibers within the gut wall (Figure 5). In addition to the ganglionated plexus, CALB^+^ nerve fibers could be observed throughout all gut layers, including the longitudinal and circular sublayers of the muscular coat (Figure 5a) and the mucosa (Figure 5b). Casually, epithelial cells with a typical bottleneck shape (enteroendocrine cells) displayed CALB reactivity. Except from Figure 5c’, we desisted from depicting the distribution patterns of SOM- and VIP-positive fibers (already published in [14,15]).

## 3. Discussion

Beyond the bare registration of numbers and proportions of CALB^+^ neurons, this study aimed to answer the question whether CALB may be a useful marker to label a particular human enteric neuron population, as has been shown for the guinea pig intestine [2]. 

### 3.1. General Distribution of CALB in the Human Enteric Plexus

Our study has extended earlier findings obtained from the human duodenum [8] by showing that CALB immunoreactivity is widely distributed throughout the myenteric and submucosal plexus of human small and large intestine. More specifically, our results obtained from analyzing the myenteric plexus of the duodenum were roughly consistent with the data of Walters et al. [8]. They found 38% of myenteric neurons to be CALB-reactive; we counted 30.6% CALB^+^ neurons. In striking contrast, they found only 13% of submucosal neurons to be CALB^+^, our proportions ranged between 72 and 82% in the two submucosal plexus of the duodenum.

An explanation for this discrepancy may be the different methodological approaches. Walters et al. [8] evaluated sections instead of wholemounts and used a light microscopic detection system (based on avidin and biotin), which may be less sensitive in contrast to our fluorescence-microscopic approach. Submucosal ganglia are composed of smaller, more tightly packed neurons displaying, in part, very weak labeling [12,13,14,15]. To detect weak CALB reactivity of these neurons, fluorescence-microscopy in wholemounts may be superior to light microscopy in sections.

As to the distribution pattern of CALB within the various gut layers, our results concur with those of Walters et al. [8] as immunoreactivity was found in all layers. Thus, the action of CALB^+^ neurons is not confined to special intestinal layers. This is in line with our finding that CALB is expressed in various different myenteric and submucosal neuron populations (see below). Hence, CALB immunoreactivity alone is not indicative for a particular neuron type. Similar to Walters et al. [8], we found occasional enteroendocrine cells in the epithelium, but this finding was not further evaluated.

### 3.2. CALB in Human Myenteric Neurons

Our NF-co-stained wholemounts revealed morphological type III neurons [18] to be, almost without exception, positive for CALB. In humans, these myenteric neurons were first described and demonstrated in silver-impregnated wholemounts [19]. They were shown to be non-nitrergic but further immunohistochemical characterization was not undertaken so far [20]. In our samples, we could only find them in wholemounts of the small intestine. Unfortunately, CALB is not suited as a selective marker for typ III neurons since it was casually found also in other, morpho-chemically different myenteric neurons. Among them there were few type II neurons [9] as well as scattered spiny and stubby type I neurons (as shown here) and small NF-negative neurons. 

Due to well-known species differences, simple transfer from the results obtained in animals is not possible. For instance, morphological type III neurons in the pig are, in contrast to human ones, nitrergic [17,21]. In the guinea pig, CALB is a marker for intrinsic primary afferent neurons [5,7,22], whereas CALB is differentially distributed in various enteric neurons of other laboratory animals [23,24,25,26]. Therefore, future studies should address the further, type-specific chemical coding of human type III neurons in order to allow conclusions as to their axonal projection pattern and, hence, their function in human small intestine. These studies will include both statistical analysis as to the proportions of type III neurons in the different small intestinal subregions and a more proper registration of the distribution pattern of their axons.

### 3.3. CALB in Human Submucosal Neurons

Most submucosal neurons belong to two cholinergic populations differing both in morphology and their other chemical coding [13]. There are non-dendritic, uniaxonal neurons displaying immunoreactivity mainly for SOM [14] and dendritic neurons reactive mainly for VIP and CALR, although the colocalization rate of both latter peptides in these neurons was almost 100% in the colon but only one third in the small intestine [15]. In the present study, CALB reactivity was found both in some SOM^+^ and in some VIP^+^ neurons. Thus, CALB labeling in the two submucosal plexus is, similar to the MP, not neuron type-specific.

### 3.4. CALB and Microbiome?

Studies in the pig [27] and mouse [28] demonstrated a correlation between experimentally altered gut microbiota and the level of calbindin expression in particular enteric neuron types. Interestingly, in these two species, CALB was expressed in neuron populations differing both morphologically and functionally, namely pig (descending) interneurons [25] versus mouse intrinsic primary afferent neurons [26]. Thus, these alterations in proportional CALB expression were not related to the changed function of an equivalent neuron type in these different species. We cannot exclude that also in human there may be a correlation between the state of the microbiome (which may be changed after tumor or other diseases and their subsequent therapies [29]) and the level of CALB expression of enteric neurons and, consequently, enteric nerve fibers. Due to the general limitation in obtaining healthy human tissues for (basic) research, this must be taken into account when interpreting data obtained from (bowel) resection samples.

## 4. Materials and Methods

### 4.1. Tissue Handling

The use of human intestinal tissues for this study was approved by the Ethics Committee of the University of Erlangen-Nuremberg (reference number 2550, 19.02.2002). The small intestinal and colonic samples derived from 26 tumor patients. Only tissue obtained from the non-tumor infiltrated borders of the resected gut segments were used. This discrimination was based on both macroscopic examination (distance from the tumor at least 10 cm) as well as on histopathological evaluation (sections stained for hematoxylin/eosin). The median age of the patients (12 female, 14 male) was 65.5 years (range between 34 and 85 years).

Intestinal segments were transported in physiological saline (pH 7.3) on ice to the laboratory. Upon arrival (up to 6 h after surgical resection), specimens were rinsed in Krebs solution at room temperature and transferred to Dulbecco´s modified Eagle´s medium (DME/F12-Ham, Sigma Chemical Company, St. Louis, MO, USA) containing 10 mg/mL antibiotic-antimycotic (Sigma, St. Louis, MO, USA), 50 µg/mL gentamycin (Sigma, St. Louis, MO, USA), 2.5 µg/mL amphotericin B (Sigma, St. Louis, MO, USA), 10% fetal bovine serum (Sigma, St. Louis, MO, USA), 4 µM nicardipine and 2.1 mg/mL NaHCO_3_, bubbled with 95% O_2_ and 5% CO_2_ at 37 °C for 1 to 2 h.

For fixation, samples were divided. The larger pieces (dedicated for wholemount preparation) were pinned onto a Sylgard-lined Petri dish and transferred to 4% formalin in 0.1 M phosphate buffered saline (PBS, pH 7.4) at room temperature for 2 to 4 h. The smaller pieces (dedicated for sections) were frozen at −70 °C in methylbutan after cryoprotection with 15% sucrose in 0.1 M PBS (2 days).

For the following immunohistochemical incubations, longitudinal muscle-myenteric plexus wholemounts and submucosal wholemounts (each about 1 × 1.5 cm) as well as cryostat sections with parallel orientation to the gut longitudinal axis were prepared.

### 4.2. Immunohistochemistry

Antibodies used for the following incubations are listed in Table 3. Three sets of wholemounts (myenteric and submucosal) were stained: the first was double stained for CALB and HU, the second set quadruple stained for CALB, CALR, SOM and VIP. The third set was dedicated for morphological analysis and similarly quadruple stained but, instead of CALR, for NF (myenteric wholemounts) or for PERI (submucosal wholemounts). This latter combination (CALB, PERI, SOM, VIP) was also applied for sections.

Incubations included the following steps: preincubation of wholemounts for 2 h (sections 1 h) in 0.05 M tris-buffered saline (TBS; pH 7.4) containing 1% bovine serum albumin (BSA), 0.5% Triton X-100, 0.05% thimerosal and 5% normal donkey serum. After rinsing in TBS for 10 min, the wholemounts were incubated in a solution containing BSA, Triton X-100, thimerosal (see above) and the primary antibodies for 72 h (4 °C; sections overnight). After an overnight rinse in TBS at 4 °C, wholemounts were incubated with secondary antibodies in the same solution as for the primary antibodies (4 h; room temperature; sections 1 h) followed by a rinse with TBS (overnight; 4 °C). 

In all specimens, we applied a lipofuscin reduction protocol after immunohistochemical labeling: incubation in ammonium acetate buffer (pH 5.0) containing 1 mM CuSO_4_ for 120 min followed by a short rinse in distilled water [20,30]. As mentioned earlier, we investigated only material that did not display any kind of neuronal autofluorescence after application of this lipofuscin reduction protocol [13].

Thereafter, specimens were mounted with TBS-glycerol (1:1; pH 8.6). Submucosal wholemounts were first mounted mucosal side up. After evaluation of the ISP, wholemounts were reversed and mounted with the outer side up for analysis of the ESP. 

With the exception of the CALB antibody (see below), negative controls for antibodies used here were carried out earlier [13,31]. Preabsorption tests for antibodies against CALR, PERI, SOM and VIP were described previously [13,15].

In this study, we tested the specificity of the CALB antibody (antigen: rat calbindin D-28k recombinant; Swant, Bellinzona, Switzerland). Preabsorption with 5-fold excess of CALB-antigen were performed overnight at 4 °C. The antigen–antibody mixtures were spun at 20,000 *g* for 20 min to sediment precipitating antigen–antibody complexes and avoid high background staining. The supernatants were then used in place of the primary antibodies. Incubation after 5-fold antigen excess resulted in no CALB staining.

### 4.3. Image Acquisition, Quantification

Wholemounts were evaluated using a confocal laser scanning microscope system (Nikon Eclipse E1000-M; Nikon Digital Eclipse C1; Tokyo, Japan) equipped with a quadruple laser configuration: a 488-nm and a 543-nm Solid-State-Laser (both from Coherent, Santa Clara, CA, USA: Sapphire 488LP, Sapphire 561-50), a 405-nm Diode-Laser (Coherent: CUBE 405-100C) and a 642-nm Diode-Laser (Melles Griot, Carlsbad, CA, USA). For reduction of unspecific background fluorescence, a BIO1-Filterset (DAPI/Cy5 for C1-Detector; AHF Analysentechnik, Tübingen, Germany) was additionally installed.

A dry objective lens (20×, numerical aperture 0.75) was used. Z-series dedicated to quantitative analysis used a zoom-factor of 2.0 (myenteric wholemounts) or 3.0 (submucosal wholemounts), z-steps were 2 µm. The figures were prepared using Volocity Demo 6.1.1 (PerkinElmer, Waltham, MA, USA), Adobe Photoshop CS6 (San Jose, CA, USA) and CorelDRAW X7 (Ottawa, ON, Canada).

In all myenteric and submucosal wholemounts, 15 ganglia or single neurons lying outside of ganglia in interganglionic nerve strands were selected randomly in a meander-like fashion, first from the inner, mucosal side of the wholemount preparation (for evaluation of the ISP), thereafter from the outer side of the wholemount (for the ESP). All counts were carried out on z-series of the ganglia, using Volocity Demo 6.1.1.

We tried to carefully discriminate neurons lying at the same x-, y- but at different z-positions to avoid double counting of neurons.

## 5. Conclusions

CALB immunoreactivity is widely distributed in the human enteric nervous system. It occurs throughout all morphological type III neurons, which we found only in the small intestinal MP. To a lesser extent, other myenteric as well as submucosal neuron types also displayed CALB reactivity. Thus, CALB alone is not an exclusive marker for human type III neurons as it is, e.g., for guinea pig type II neurons (IPANs). Beyond that, we cannot rule out a correlation between a possibly altered microbiome (after tumors or other diseases and their subsequent therapies) and the CALB expression of enteric neurons.

## Figures and Tables

**Table 1:** Mean values ± standard deviations of neuron numbers and proportions of means in 15 ganglia per wholemount (i.e., per subject) that were immunolabeled for the pan-neuronal marker HU alone as well as for both HU and CALB.

Segment	Plexus	HU	HU CALB	%
*n* = Patients				CALB
Duodenum *n* = 3	MP	539.0 ± 23.9	165.0 ± 11.8	30.6
	ESP	164.3 ± 20.1	119.3 ± 14.8	72.6
	ISP	199.0 ± 35.4	164.3 ± 16.6	82.6
Jejunum *n* = 3	MP	367,7 ± 36.1	92.3 ± 11.3	25.1
	ESP	138.3 ± 22.8	119.3 ± 16.8	86.3
	ISP	182.7 ± 31.2	151.7 ± 20.3	83
Ileum *n* = 3	MP	435.3 ± 36.5	159.7 ± 15.6	36.7
	ESP	128.0 ± 29.5	72.7 ± 15.0	56.8
	ISP	185.7 ± 22.0	143.7 ± 18.7	77.4
**Σ Small intestine** ***n* = 9**	**MP**	**447.3** ± 39.9	**139.0** ± 17.2	**31.1**
	**ESP**	**143.5** ± 30.7	**103.8** ± 18.1	**72.3**
	**ISP**	**189.1** ± 36.9	**153.2** ± 22.8	**81**
Ascending colon *n* = 5	MP	413.4 ± 38.9	101.4 ± 10.6	24.5
	ESP	120.2 ± 20.6	112.2 ± 16.1	93.3
	ISP	220.0 ± 31.3	212.6 ± 19.7	96.6
Transverse colon *n* = 4	MP	514.0 ± 36.6	156.7 ± 14.0	30.5
	ESP	202.5 ± 16.0	188.5 ± 16.9	93.1
	ISP	203.7 ± 27.4	197.7 ± 19.7	97
Descending colon *n* = 3	MP	430.3 ± 36.6	91.0 ± 21.0	21.1
	ESP	181.3 ± 29.9	164.3 ± 20.1	90.6
	ISP	217.7 ± 16.4	201.3 ± 18.8	92.5
Sigmoid colon *n* = 5	MP	418.8 ± 22.2	96.4 ± 16.9	23
	ESP	173.4 ± 19.6	163.8 ± 14.2	94.5
	ISP	271.2 ± 24.4	253.8 ± 18.0	93.6
**Σ Large intestine** ***n* = 17**	**MP**	**441.6** ± 40.0	**111.1** ± 24.2	**25.2**
	**ESP**	**166.0** ± 33.5	**154.5** ± 22.9	**93.1**
	**ISP**	**230.8** ± 30.7	**219.2** ± 20.6	**95**

MP = myenteric plexus; ESP = external submucosal plexus; ISP = internal submucosal plexus.

**Table 2:** Numbers (means ± standard deviations) of neurons stained for CALB in 15 ganglia per wholemount (i.e., per subject) and proportions of CALB neurons without co-staining or displaying colocalization with other markers, respectively.

Segment *n* = Patients	Plexus	CALB	CALB Only	CALB VIP	CALB CALR	CALB	CALB SOM	CALB
		Number				CALR		SOM
		Σ				VIP		CALR
Duodenum *n* = 3	MP	136.7 ± 19.0	96.90%	0.20%	1.20%	0.00%	0.20%	1.20%
	ESP	68.0 ± 9.8	9.60%	3.80%	0.00%	53.80%	31.70%	0.90%
	ISP	85.3 ± 14.6	0.60%	6.40%	12.20%	41.70%	39.10%	0.00%
Jejunum *n* = 3	MP	166.3 ± 22.0	83.00%	6.00%	4.80%	4.20%	0.60%	1.00%
	ESP	79.3 ± 13.1	1.40%	1.40%	3.60%	65.90%	21.00%	2.90%
	ISP	86.0 ± 12.0	0.00%	0.60%	5.10%	67.10%	17.70%	9.50%
Ileum *n* = 3	MP	159.0 ± 18.8	79.90%	6.50%	7.30%	0.60%	3.60%	1.00%
	ESP	70.0 ± 12.4	1.40%	0.00%	7.10%	81.40%	9.50%	0.50%
	ISP	95.3 ± 14.8	0.30%	0.70%	3.80%	85.00%	10.10%	0.00%
**Σ Small intestine** ***n* = 9**	**MP**	**154.0 ± 24.2**	**86.00%**	**4.50%**	**4.60%**	**1.70%**	**1.50%**	**1.10%**
	**ESP**	**72.4 ± 14.8**	**3.30%**	**1.30%**	**4.40%**	**70.30%**	**18.10%**	**1.30%**
	**ISP**	**88.9 ± 16.0**	**0.30%**	**2.20%**	**6.30%**	**69.00%**	**19.70%**	**2.50%**
Ascending	MP	152.8 ± 18.0	65.40%	12.80%	13.70%	7.10%	0.60%	0.00%
colon	ESP	91.0 ± 16.8	1.30%	11.00%	5.90%	77.80%	3.70%	0.00%
*n* = 5	ISP	160.4 ± 22.2	2.00%	14.50%	2.20%	72.10%	8.70%	0.20%
Transverse colon *n* = 4	MP	189.0 ± 19.7	72.90%	14.30%	7.90%	4.60%	0.00%	0.30%
	ESP	161.2 ± 17.9	1.80%	0.40%	8.90%	85.30%	3.20%	0.10%
	ISP	184.0 ± 20.0	1.80%	0.30%	3.10%	87.50%	4.20%	2.40%
Descending colon *n* = 3	MP	65.0 ± 12.0	61.00%	31.30%	4.10%	3.10%	0.00%	0.50%
	ESP	76.3 ± 14.9	1.70%	2.20%	5.20%	90.40%	0.40%	0.00%
	ISP	84.7 ± 14.0	10.60%	4.70%	3.50%	79.10%	2.00%	0.00%
Sigmoid colon *n* = 5	MP	99.4 ± 8.6	84.30%	2.40%	10.90%	1.60%	0.40%	0.40%
	ESP	101.8 ± 17.6	4.70%	15.70%	5.10%	72.30%	2.20%	0.00%
	ISP	140.6 ± 18.1	0.80%	17.10%	2.30%	73.40%	6.10%	0.00%
**Σ Large intestine** ***n* = 17**	**MP**	**130.1 ± 22.0**	**71.80%**	**12.60%**	**10.30%**	**4.60%**	**0.30%**	**0.20%**
	**ESP**	**108.6 ± 19.3**	**2.50%**	**7.50%**	**6.70%**	**80.50%**	**2.70%**	**0.00%**
	**ISP**	**146.8 ± 24.3**	**2.50%**	**10.00%**	**2.70%**	**77.70%**	**6.00%**	**0.80%**

Neurons co-labeled for all four markers or for CALB, SOM and VIP were only exceptionally observed and are not included in the table but mentioned in the text. (MP = myenteric plexus; ESP = external submucosal plexus; ISP = internal submucosal plexus).

**Table 3:** Antisera.

***Primary Antisera***			
**Antigen**	**Host**	**Dilution**	**Source**
Calbindin D28k	Rabbit	1:1500	CB-38; Swant; Switzerland
HUC/D	Mouse	1:50	A21271; Thermo Fisher Scientific; Germany
NF 200	Mouse	1:200	N0142; Sigma, Germany
Peripherin	Goat	1:200	sc-7604; Santa Cruz; Germany
Calretinin	Mouse	1:1000	M7245; Dako, Germany
Somatostatin (YC7)	Rat	1:200	sc-47706; Santa Cruz, Germany
VIP	Guinea-pig	1:500	T-5030; Dianova, Germany
***Fluorescence Tags for Secondary Antisera***			
Alexa Fluor 555	Donkey anti-rabbit	1:1000	A31572; Thermo Fisher Scientific, Germany
Alexa Fluor 488	Donkey anti-mouse	1:1000	A21202; Thermo Fisher Scientific, Germany
Alexa Fluor 488	Donkey anti-goat	1:1000	A11055; Thermo Fisher Scientific, Germany
Dy-Light 647	Donkey anti-rat	1:1000	712-605-153; Dianova, Germany
Dy-Light 405	Donkey anti-guinea pig	1:200	706-475-148; Dianova, Germany

## Abbreviations

CALB	Calbindin
CALR	Calretinin
ESP	External Submucosal Plexus
HU	Neuronal Protein HUC/D
IPAN	Intrinsic Primary Afferent Neuron
ISP	Internal Submucosal Plexus
MP	Myenteric Plexus
NF	Neurofilament
PERI	Peripherin
PBS	Phosphate Buffered Saline
SOM	Somatostatin
TBS	Tris-Buffered Saline
VIP	Vasoactive Intestinal Peptide
